# Sex-specific relationships among iron status biomarkers, athletic performance, maturity, and dietary intakes in pre-adolescent and adolescent athletes

**DOI:** 10.1186/s12970-019-0306-7

**Published:** 2019-09-18

**Authors:** Marni E. Shoemaker, Zachary M. Gillen, Brianna D. Mckay, Nicholas A. Bohannon, Sydney M. Gibson, Karsten Koehler, Joel T. Cramer

**Affiliations:** Department of Nutrition and Health Sciences, University of Nebraska-Lincoln, Ruth Leverton Hall, Lincoln, NE 68583 USA

**Keywords:** Youth athletes, Exercise, Nutrition, Athletic performance, Iron

## Abstract

**Background:**

The purpose of this study was to examine relationships among biomarkers of iron status, athletic performance, growth and development, and dietary intakes in pre-adolescent and adolescent male and female athletes.

**Methods:**

Two-hundred and forty-nine male (*n* = 179) (mean ± standard deviation for age = 12.0 ± 2.1 years, height = 156.3 ± 13.9 cm, and weight = 49.1 ± 16.5 kg) and female (*n* = 70) (12.0 ± 2.2 years, 152.4 ± 12.3 cm, 45.3 ± 14.5 kg) athletes volunteered for capillary blood sample, anthropometric, athletic performance, and dietary intake assessments. Outcomes included maturity offset from peak height velocity, percent body fat, estimated muscle cross-sectional areas, vertical jump height (VJ), broad jump distance (BJ), pro-agility time (PA), L-cone time, 20-yard dash time (20YD), power push up (PPU) force, dietary intakes, and ferritin, soluble transferrin receptor (sTfR), and hemoglobin (Hb) concentrations.

**Results:**

Athletic performance was consistently correlated with Hb in males (*r* = .237–.375, *p* < 0.001–0.05) and with sTfR (*r* = .521–.649, *p* < 0.001–0.004) and iron intake (*r* = .397–.568, *p* = 0.001–0.027) in females. There were no relationships between dietary intakes and ferritin, sTfR, or Hb (*p* > 0.05). After partialing out age and height, VJ, PA, LC, and 20YD remained correlated with Hb in males (|*r*_Hb,y.Age_| = .208–.322, *p* = 0.001–0.041; |*r*_Hb,y.Height_| = .211–.321, *p* = 0.001–0.038). After partialing out iron intake, PA and LC remained correlated with sTfR in females (|*r*_sTfR,y.ironintake_| = .516–.569, *p* = 0.014–0.028).

**Conclusions:**

Iron status biomarkers demonstrated sex-specific relationships with anaerobic exercise performance in youth athletes, which may be more dependent on maturity status and dietary intake than age. Moderate relationships between sTfR and athletic performance in adolescent female athletes emphasizes the importance of iron intake in this demographic.

## Background

Iron plays important roles for athletic performance, including red blood cell production, oxygen transport, and electron transport during oxidative phosphorylation [1–4]. Biomarkers used to measure iron status in athletes have included ferritin, soluble transferring receptor (sTfR), and hemoglobin (Hb) concentrations [5–7]. Previous studies have demonstrated positive associations between athletic performance measurements and ferritin and Hb concentrations [8–11], while sTfR concentrations have been inversely related to exercise [12]. Therefore, exercise and athletic performance is impacted by iron status, which suggests that maintaining adequate intakes of dietary iron may be important for athletes.

Dietary iron requirements for children are also important for healthy growth and development. Children have increased dietary iron requirements due to high growth rates of bone and muscle, increased plasma volumes, onset of menarche in females, and often inadequate consumption of dietary iron [5, 13, 14]. Given the increased popularity of competitive youth sports [15], youth athletes may exhibit a particularly high demand for dietary iron intake when considering both growth and development and athletic performance requirements.

An early study by Cullumbine [8] showed that speed and strength were related to Hb concentrations in adolescent males, but these relationships did not exist for females of the same age. The authors also reported greater performance scores and Hb concentrations in 14–20-year old males compared to females. Nearly 60 years later, Gracia-Marco and colleagues [10] reported remarkably similar relationships between Hb and both cardiorespiratory and muscular fitness in 12.5–17.5-year old males, but not females. Mechanisms exist for how oxygen transport and utilization can be related to anaerobic exercise performance, which may also provide a theoretical construct for relationships between Hb and anaerobic performance. For example, the use of aerobic metabolism is suggested to be predominant during adolescence, as measured levels of oxidative enzymes were higher in young males and females compared to adults [16, 17]. Since children rely more heavily on myoglobin-rich, oxidative fibers [17, 18], the oxygen carrying capacity of Hb or myoglobin may be more influential during anaerobic performance in children. Furthermore, the resynthesis of creatine phosphate within the mitochondria of skeletal muscle is oxygen-dependent [19, 20]. Given that 49–57% of children in the United States participate in team and individual sports [21] and nearly all those sports are anaerobic in nature, evaluating relationships among iron status and anaerobic performance in youth may appropriately reflect their state of health and physical activity.

While previous studies have demonstrated relationships between athletic performance and ferritin [9], sTfR [12], and Hb [11] in adult athletes, there is a lack of research directly relating concentrations reflecting iron status to athletic performance in young athletes. Overall, these previous studies [8–11] have raised questions about the relationships between Hb concentrations and aerobic versus anaerobic exercise performance as well as the potential value of relationships among ferritin and sTfR concentrations and exercise performance in females. However, results in adults cannot be extrapolated to pre-adolescent and adolescent athletes due to differences in energy utilization [22]. Previous studies examining young athletes reported prevalence of iron deficiency and anemia [5, 23], yet few studies [8, 24] examined direct relationships with athletic performance in this younger population.

Athletic differentiation between males and females is thought to occur during adolescence [25, 26]; thus, studying the role of iron in relation to athletic performance in adolescents by sex may also provide insight regarding dietary recommendations for optimizing their health. Therefore, the purpose of the present study was to examine the relationships among biomarkers of iron status, athletic performance, growth and development, and dietary intakes of young male and female athletes. It was hypothesized that while there would be a positive relationship among iron status and athletic performance based on previous studies, [8, 10, 11] sex differentiations pertaining to individual biomarkers would emerge based on differences in growth and development of young males and females.

## Methods

### Study design

A cross-sectional design was used to quantify relationships among athletic performance tests, measures of dietary intake, and hematological biomarkers of iron status in male and female adolescent athletes ages 5 to 18 years old.

### Subjects

Male (*n* = 179) and female (*n* = 70) adolescent athletes (*n* = 249 total) volunteered for this study. Subjects were 5–18 years old and actively participating in school- or club-sponsored sports that held regular practices. Sports included baseball, basketball, cheerleading, cross country, dance, equestrian, football, golf, gymnastics, hockey, lacrosse, martial arts, rugby, soccer, softball, speed/power/agility training, swimming/diving, tennis, track and field, trap shooting, volleyball, weightlifting, and wrestling. Participants completed the Physical Activity Readiness Questionnaire for everyone (PAR-Q+ 2015), [27] that consists of general health questions to determine if the participant is safe to engage in physical activity. This study was approved by the University of Nebraska-Lincoln Institutional Review Board for the protection of human subjects (IRB # 20160616246EP, Title: Youth Combine Testing, approval date: June 24, 2016). Each participant signed an approved youth assent form if they were 7–18 years old, and if the participant was 5–6 years old, verbal assent was obtained. One parent or legal guardian of each participant signed an approved informed consent document.

### Anthropometrics and body composition

Height (cm) and weight (kg) were measured using a beam scale with attached stadiometer (Mechanical Column Scale & Stadiometer, Seca gmbh & co. kg, Hamburg, Germany). Seated height was measured to calculate maturity offset to predict peak height velocity (PHV) [28]. A maturity offset of less than − 0.5 years from PHV was considered pre-adolescent; − 0.5 to + 0.5 years from PHV was considered adolescent; and greater than + 0.5 years from PHV was considered post-adolescent [28, 29]. Body composition measurements included percent body fat (BF%), arm estimated cross-sectional area (eCSA), and thigh eCSA. Skinfold measurements were taken with a Lange caliper (Model 68,902, Cambridge Scientific Industries, Inc., Cambridge, MD, USA) and were used to calculate BF%. Skinfold measurements were taken on the right side of the body at the triceps (vertical fold in the middle of the upper arm, midway between the acromion and olecranon process) and anterior suprailiac (diagonal fold immediately superior to the anterior superior iliac spine) for males, and the triceps, suprailiac (diagonal fold 1 cm above the anterior superior iliac crest), and subscapula (diagonal fold 2 cm below the inferior angle of the scapula) for females. All skinfolds were recorded to the nearest 0.5 mm [30] and were entered into equations established by Housh et al. [31] and Brozek et al. [32] to estimated body density and BF%, respectively.

Arm and thigh circumferences were measured using a Gulick measurement tape (Baseline® measurement tape with Gulick attachment, Fabrication Enterprises, White Plains, NY) and recorded to the nearest 0.1 cm. Arm circumference and triceps skinfold were used to calculate arm eCSA, while thigh circumference and thigh skinfold (vertical pinch at the mid-point of the anterior surface of the thigh, halfway between the patella and inguinal fold) were used to calculate thigh eCSA using procedures described by Moritani and deVries [33].

### Athletic performance testing

Detailed procedures of all athletic performance testing measurements are described by Gillen et al. [34]. Testing was conducted with similar methodology and equipment as the basic tests performed at the National Football League (NFL) scouting combine. Tests included the vertical jump (VJ), broad jump (BJ), pro-agility (PA), L-cone (LC), 20-yard dash (20YD) and power push up (PPU). The VJ was an assessment of vertical jumping performance measured with a Vertec (Sports Imports, Freestanding Vertec Jump Trainer, Hilliard, OH, USA) and was calculated as the difference between standing reach and the highest jump recorded (cm). BJ assessed horizontal jumping performance as the distance between the starting line and the heel of the subject closest to the starting line (cm). The two agility drills, PA and LC, and the 20YD, were measured in seconds (s) using a digital, laser beam actuated timing gate with motion start (Brower Timing Systems, Brower TC Motion Start Timer, Knoxville, TN, USA). Splits were recorded at 5 and 10 yards during the 20YD.

### Dietary intake assessments

Among the total sample (*n* = 249), 39% (*n* = 97; male, *n* = 66; female, *n* = 31) also completed a 24-h dietary recall administered online using the Automated Self-Administered 24-h (ASA24®) Dietary Recall System. If the participant was less than 14 years old, the recall was administered to a parent or legal guardian for completion. Participants were prompted with detailed questions regarding food intake with regard to serving sizes and composition of food choices. Total energy (kcal·d^− 1^), carbohydrate (g·d^− 1^), protein (g·d^− 1^), fat (g·d^− 1^), and iron (mg·d^− 1^) intakes were quantified and reported from the ASA24®.

### Biomarkers of Iron status

Capillary blood samples of 400 μL were collected in microvettes (Microvette® 200 μL, K3 EDTA, violet US code; 10.8 mm × 46.6 mm) to analyze ferritin and sTfR. Human alpha 1-acid glycoprotein (AGP) was assessed to determine inflammatory status of the participant to correct ferritin concentrations if falsely elevated [35]. Enzyme-linked immunosorbent assay (ELISA) kits were used to assess concentrations of ferritin (μg·L^− 1^; *n* = 118; males, *n* = 94; females, *n* = 24) (ELISA kit Ramco Labs), sTfR (nmol·L^− 1^; *n* = 105; males, *n* = 76; females, *n* = 29) (Quantikine IVD ELISA Kit, R&D Systems), and AGP (μmol ·L^− 1^; *n* = 40; males, *n* = 39; females, n = 1) (ELISA kit, R&D Systems). AGP was quantified in a lower sample, since after the first 40 assays, none exhibited a high enough inflammatory status to warrant a correction of ferritin. Assay procedures were followed per kit instructions and absorbance was read at 500 and 650 nm for ferritin and 450 and 540 nm for sTfR and AGP. Hemoglobin (Hb) concentration (g·L^− 1^) was assessed on site during the athletic performance testing with a handheld hemoanalyzer (AimStrip®*Hb* Hemoglobin meter, Germaine Laboratories, Inc.) in 51% of the total sample (*n* = 128; male, *n* = 100; female, *n* = 28).

### Statistical analyses

Means and standard deviations for anthropometrics, performance measurements, dietary intakes, and biomarkers of iron status were calculated in a spreadsheet software program (Microsoft Excel 2017, version 16.10) (Table 1). Exploratory data analysis for outliers was performed using the Tukey procedure [36]. Independent-samples t-tests (with unequal variances assumed) were used to compare the mean values of males versus females (Table 1). A Pearson product moment correlation analysis was performed with and without outliers, among all 7 descriptive and anthropometric variables, 6 performance variables, 5 dietary intake variables, and 3 biomarkers of iron status for all athletes (Table 2) and separated by males and females. Correlation coefficients were evaluated qualitatively according to Mukaka [37]: 0.00 to 0.30 = negligible; 0.30 to 0.50 = low; 0.50 to 0.70 = moderate; 0.70 to 0.90 = high; 0.90 to 1.00 = very high. For significant collinear relationships among anthropometrics, athletic performance, dietary intakes, and iron status biomarkers, first-order partial correlations (r_xyz_) were calculated to partial out collinear influences. All statistical analyses were performed using IBM SPSS Statistics for Macintosh, Version 24 (IBM Corp., Chicago, IL, USA.) An alpha of *p* ≤ 0.05 was considered statistically significant for all correlations and comparisons.

**Table 1:** Demographics, anthropometrics, athletic performance scores, dietary intakes and biomarkers of iron status

	Composite (*n* = 249)	Males (*n* = 179)	Females (*n* = 70)	Outliers (*n* = 16)
Age (y)	12.0 ± 2.1 (*n* = 249)	12.0 ± 2.1 (*n* = 179)	12.0 ± 2.2 (*n* = 70)	
Maturity Offset (y)	−1.3 ± 1.9 (*n* = 249)	−1.7 ± 1.7 (*n* = 179)^*^	−0.1 ± 1.8 (*n* = 70)	
Height (cm)	155.2 ± 13.6 (*n* = 249)	156.3 ± 13.9 (*n* = 179)^*^	152.4 ± 12.3 (*n* = 70)	
Weight (kg)	48.0 ± 16.0 (*n* = 249)	49.1 ± 16.5 (*n* = 179)	45.3 ± 14.5 (*n* = 70)	120.6 kg
Body Fat (%)	20.2 ± 6.5 (*n* = 244)	19.7 ± 6.7 (*n* = 175)	21.5 ± 5.9 (*n* = 69)	47.9%
Arm eCSA (cm^2^)	14.3 ± 6.9 (*n* = 246)	15.6 ± 7.3 (*n* = 176)^*^	11.2 ± 4.6 (*n* = 70)	45.74 cm^2^
Thigh eCSA (cm^2^)	80.6 ± 31.5 (*n* = 245)	83.3 ± 33.0 (*n* = 175)^*^	73.7 ± 26.3 (*n* = 70)	
Vertical Jump (cm)	40.2 ± 9.4 (*n* = 246)	41.7 ± 9.6 (*n* = 177)^*^	36.4 ± 7.4 (*n* = 69)	
Broad Jump (cm)	168.6 ± 30.2 (*n* = 247)	172.9 ± 30.6 (*n* = 178)^*^	157.3 ± 26.2 (*n* = 69)	
Pro-Agility (s)	5.8 ± 0.6 (*n* = 247)	5.7 ± 0.6 (*n* = 177)^*^	5.9 ± 0.5 (*n* = 70)	8.76 s
L Cone (s)	9.4 ± 0.9 (*n* = 245)	9.3 ± 1.0 (*n* = 176)^*^	9.7 ± 0.8 (*n* = 69)	15.0 s
20 Yard Dash (s)	3.7 ± 0.5 (*n* = 248)	3.7 ± 0.5 (*n* = 178)^a^	3.8 ± 0.4 (*n* = 70)	5.98, 6.79 s
Power Push Up (N)	170.6 ± 84.1 (*n* = 246)	185.5 ± 90.0 (*n* = 177)^*^	132.6 ± 51.3 (*n* = 69)	583, 601 N
Energy Intake (kcals·d^− 1^)	2052 ± 711 (*n* = 97)	2158 ± 749 (*n* = 66)^*^	1827 ± 568 (*n* = 31)	
Carbohydrates (g·d^− 1^)	244 ± 89 (*n* = 97)	256 ± 89 (*n* = 66)^*^	217 ± 83 (*n* = 31)	
Protein (g·d^− 1^)	90 ± 38 (*n* = 97)	98 ± 41 (*n* = 66)^*^	74 ± 25 (*n* = 31)	
Fat (g·d^−1^)	82 ± 37 (*n* = 97)	84 ± 39 (*n* = 66)	76 ± 34 (*n* = 31)	
Iron (mg·d^− 1^)	16.5 ± 9.7 (*n* = 97)	17.9 ± 10.9 (*n* = 66)^*b^	13.5 ± 5.5 (*n* = 31)	46.0, 44.8, 55.0, 62.0 mg·d^− 1^
Hemoglobin (g·L^− 1^)	113 ± 16 (*n* = 128)	114 ± 16 (*n* = 100)	112 ± 19 (*n* = 28)	
Ferritin (μg·L^− 1^)	24.0 ± 15.0 (*n* = 118)	25.3 ± 16.2 (*n* = 94)^*^	18.6 ± 7.3 (*n* = 24)	
sTfR (nmol·L^− 1^)	22.1 ± 6.4 (*n* = 105)	21.9 ± 6.8 (*n* = 76)	22.8 ± 5.5 (*n* = 29)	38.8, 44.5, 66.7 nmol·L^− 1^

Values are means ± standard deviations (SD)

^*^Indicates a significant difference between the mean values of males versus females (*p* ≤ 0.05) with outliers included

^a^Indicates a significant difference after removal of outliers. ^b^Indicates difference became non-significant after removal of outliers

**Table 2:** Pearson product moment correlation coefficient matrix among all variables for composite sample of young athletes

	Age		Maturity Offset		Height		Weight		BF%		Arm eCSA		Thigh eCSA		VJ		BJ		PA		LC		20YD		PPU		Hb		Ferritin		sTfR		Energy Intake		Protein	Fat		Carbohydrate		
*N* pairs	249		249		249		249		244		246		245		246		247		247		245		248		246		128		118		105		97		97	97		97		
	M	F	M	F	M	F	M	F	M	F	M	F	M	F	M	F	M	F	M	F	M	F	M	F	M	F	M	F	M	F	M	F	M	F	M	F	M	F	M	F
*N* pairs	179	70	179	70	179	70	179	70	175	69	176	70	175	70	177	69	178	69	177	70	176	69	178	70	177	69	100	28	94	24	76	29	66	31	66	31	66	31	66	31
Maturity Offset	.875^a^																																							
	.946^a^	.972^a^																																						
Height	.837^a^		.802^a^																																					
	.839^a^	.859^a^	.927^a^	.945^a^																																				
Weight	.690^a^		.714^a^		.800^a^																																			
	.669^•^	.682^a^	.841^a^	.779^a^	.812^a^	.748^a^																																		
BF%	−.103		−.034		−.056		.352^a^																																	
	−.260^a^	.333^a^	−.173^ac^	.425^a^	−.166^a^	.378^a^	.250^a^	.775^a^																																
Arm eCSA	.459^a^		.347^a^		.506^a^		.463^a^		−.388^a^																															
	.507^a^	.386^a^	.550^a^	.430^a^	.519^a^	.398^a^	.416^a^	.639^a^	−.508^a^	.251^ac^																														
Thigh eCSA	.666^a^		.632^a^		.704^a^		.716^a^		−.073		.591^a^																													
	.667^a^	.696^a^	.754^a^	.747^a^	.692^a^	.726^a^	.721^a^	.681^a^	−.140^a^	.245^ac^	.573^a^	.638^a^																												
VJ	.636^a^		.447^a^		.586^a^		.321^a^		−.496^a^		.566^a^		.537^a^																											
	.674^a^	.616^a^	.627^a^	.613^a^	.575^a^	^.^593^a^	.303^a^	.321^a^	−.585^a^	−.097	.569^a^	.336^a^	.509^a^	.593^a^																										
BJ	.639^a^		.452^a^		.571^a^		.290^a^		−.453^a^		.530^a^		.526^a^		.844^a^																									
	.656^a^	.664^a^	.589^a^	.669^a^	.527^a^	.683^a^	.261^a^	.323^a^	−.544^a^	.083	.558^a^	.225	.499^a^	.564^a^	.878^a^	.663^a^																								
PA	−.635^a^		−.434^a^		−.512^a^		−.184^a^		.496^a^		−.401^a^		−.382^a^		−.820^a^		−.803^a^																							
	−.621^a^	−.716^a^	−.523^a^	−.673^a^	−.465^a^	−.619^a^	−.144^b^	−.249^a^	.608^a^	.069	−.423^a^	−.127	−.323^a^	−.509^a^	−.840^a^	−.713^a^	−.834^a^	−.662^a^																						
LC	−.606^a^		−.410^a^		− 493^a^		−.201^a^		.473^a^		−.433^a^		−.369^a^		−.762^a^		−.748^a^		.920^a^																					
	−.600^a^	−.654^a^	−.505^a^	−.610^a^	−.462^a^	−.545^a^	−.165^a^	−.251^a^	.581^a^	.055	−.462^a^	−.134	−.314^a^	.479^a^	−.761^a^	−.726^a^	−.773^a^	−.612^a^	.926^a^	.885^a^																				
20YD	−.593^a^		−.428^a^		−.485^a^		−.151^a^		.470^a^		−.349^a^		−.345^a^		−.754^a^		−.734^a^		.874^a^		.811^a^																			
	−.569^a^	−.661^a^	−.476^a^	−.619^a^	−.399^a^	−.595^a^	−.100^b^	−.255^ac^	.600^a^	.066	−.386^a^	−.129	−.289^a^	−.471^a^	−.767^a^	−.721^a^	.777^a^	−.600^a^	.885^a^	.847^a^	.843^a^	.709^a^																		
PPU	.585^a^		.478^a^		.598^a^		.660^a^		−.041		.508^a^		.664^a^		.485^a^		.520^a^		−.399^a^		−.396^a^		−.346^a^																	
	.680^a^	.381^a^	.754^a^	.390^a^	.650^a^	.343^a^	.731^•^	.350^a^	−.039	.158	.497^a^	.239^a^	.719^a^	.343^a^	.491^a^	.190	.482^a^	.423^a^	−.397^a^	−.232	−.393^a^	−.216	−.373^a^	−.157																
Hb	.184^a^		.139		.194^a^		.159		−.037		.075		.086		.247^a^		.214^a^		−.230^a^		−.228^a^		−.204^a^		.221^a^															
	.202^a^	.145	.188	.157	.219^a^	.101	.112	.283	−.147	.288	.031	.205	.102	.026	.331^a^	−.002	.277^a^	.037	−.317^a^	.064	−.284^a^	−.034	−.375^a^	.177	.237^a^	.146														
Ferritin	−.016		−.027		.099		.252^a^		.278^a^		−.049		−.052		−.137		−.166		.181		.141		.181^a^		.070		.065													
	.019	−.011	.045	.081	.063	.195	.246^a^	.345	.323^a^	.257	−.137	.386	−.087	.130	−.201	−.184	−.272^a^	.150	.222^a^	.360	.188	.313	.223^a^	.200	.033	−.034	.000	.606^a^												
sTfR	.009^b^		.099		.050		.061		.005		.048		.046		−.093^b^		−.047		.073^b^		.088^b^		.070^b^		−.011		.033		−.223^ac^											
	.126	−.295	.196	−.240	.132	−.182	.093	−.001	−.067^b^	.200	.125	−.183	.132	−.215	.084	−.562^a^	.068	−.308	−.079	.522^a^	−.092	.649^a^	−.132	.521^a^	−.001	.126	.023	.086	−.262^ac^	.228										
Energy Intake	.047		−.054		.088		−.052		−.298^a^		.182		.112		.276^a^		.253^a^		−.266^a^		−.252^a^		−.205^a^		.088		−.080		−.053		−.272^a^									
	.051	.015	.067	−.045	.084	−.041	−.068	−.250	−.281^a^	−.344	.162	−.076	.139	−.084	.263^a^	.188	.187	.240	−.265^a^	−.102	−.186	−.233	−.197	−.146	−.101	−.326	−.133	.040	−.101	.236	−.297	−.278								
Protein	.153		.041		.247^a^		.195		−.162		.325^a^		.288^a^		.312^a^		.260^a^		−.237		−.217^a^		−.193^b^		.208^ac^		.017		.131		−.222		.763^a^							
	.251^a^	−.132	.285^a^	−.141	.280^b^	−.46	.202	−.169	−.138	−.232	.295^ac^	.020	.352^a^	−.096	.290^a^	.213	.201	.139	−.213	−.054	−.144	−.142	−.162	−.151	.215	−.326	−.030	.143	.093	.244	−.234	−.198	.789^a^	.577^a^						
Fat	.045		.003		.076		−.039		−.210^a^		.071		.122		.174		.154		−.179		−.140		−.133		.096		−.194		−.117		−.218		.895^a^		.700^a^					
	.035	.056	.061	.002	.080	.002	−.038	−.162	−.198	−.231	.055	−.033	.179	−.103	.164	.148	.112	.174	−.173	−.121	−.093	−.150	−.105	−.143	.130	−.186	−.185	−.238	−.093	−.151	−.195	−.312	.916^a^	.847^a^	.731^a^	.623^a^				
Carbo-hydrate	−.029		−.132		−.011		−.170		−.340^a^		.144		.024		.245^a^		.243^a^		−.253^a^		−.271^a^		−.199^b^		−.018		.010		−.052		−.248^b^		.839^a^		.425^a^		.551^a^			
	−.066	.019	−.071	−.031	.043	−.051	−.222	−.229	−.342^a^	−.324	.139	−.129	−.056	−.024	.249^a^	.120	.180	.222	−.277^a^	−.054	−.219	−.231	−.229	−.080	−.032	−.288	−.094	.237	−.162	.454	−.341^a^	−.125	.852^a^	.781^a^	.469^a^	.114	.614^a^	.357^a^		
Iron	.138		.032		.153		−.029		−.274^a^		.091^b^		.162		.312^a^		.215^ac^		−.220^ac^		−.224^ac^		−.242^ac^		.001		.074		.112		−.240		.414^a^		.348^a^		.271^a^		.440^a^	
	.061	.436^a^	.069	.387^a^	.086	.323	−.109	.162	−.264^ac^	−.314	−.011	.363^a^	.072	.535^a^	.225	.565^a^	.078	.568^a^	−.130	−.397^a^	−.177	−.465^a^	−.155	−.466^a^	−.056	−.130	.030	.223	.055	.365	−.217	−.341	.392^a^	.377^a^	.329^a^	.155	.288^a^	.126	.414^a^	.476^a^

The top row indicates the correlation with all athletes and the bottom row indicates the correlation separated by male (M) on the left side and female (F) on the right side

^a^Correlation is significant at the 0.05 level with outliers included; ^b^Correlation became significant with removal of outliers; ^c^Correlation became non-significant with removal of outliers

## Results

Outliers (*n* = 16) were identified for weight (*n* = 1), BF% (*n* = 1), arm eCSA (*n* = 1), PA (*n* = 1), LC (*n* = 1), 20YD (*n* = 2), PPU (*n* = 2), iron (*n* = 4), and sTfR (*n* = 3), and the values for each outlier are reported in Table 1. The independent samples t-tests showed significant differences between males and females for maturity offset, height, arm eCSA, thigh eCSA, VJ, BJ, PA, LC, PPU, ferritin, energy intake, protein, carbohydrates, and iron (*p* < 0.001–0.048). With outliers removed, the sex difference in 20YD time became significant (*p* = 0.041), while the sex difference in iron intake became non-significant (*p* = 0.104) (Table 1).

Table 2 illustrates the significant interrelationships (*p* ≤ 0.05) among the anthropometric measurements in the composite sample and separated by sex. Specifically, age, maturity offset, height, weight, and thigh eCSA demonstrated moderate to very high intercorrelations. Arm eCSA showed low intercorrelations among females and moderate intercorrelations among males. Therefore, age, maturity offset, height, weight, and thigh eCSA were interpreted to collectively reflect growth and development in females, while arm eCSA was added to the same group of variables to reflect growth and development in males. BF% showed mostly negligible to low intercorrelations and was subsequently excluded from growth and development (Table 2).

Similarly, the VJ, BJ, PA, LC, and 20YD measurements were consistently interrelated at a significant level (*p* ≤ 0.05) within the composite sample as well as the separate male and female correlation matrices. The direction of the correlation reflected the measurement (distance, time, or power) such that better performance occurred with greater distance (VJ or BJ) and greater power (PPU), whereas better performance occurred with lower time-scored variables (PA, LC, and 20YD). Intercorrelations among VJ, BJ, PA, LC, and 20YD were all high or very high, except for BJ in the females, which exhibited moderate intercorrelations. Therefore, these variables were interpreted to collectively reflect athletic performance (Table 2). PPU scores exhibited negligible to low intercorrelations among the other variables and was subsequently excluded from the grouping.

From the ASA24®, energy, carbohydrate, protein, fat, and iron intakes demonstrated consistent, but not uniform, significant intercorrelations (*p* ≤ 0.05). Iron exhibited mostly negligible to low relationships among the other dietary intakes. By virtue of how these variables were collected and reported, all were collectively interpreted as dietary intakes; however, they were also considered individually for relationships with growth and development, athletic performance, and biomarkers of iron status (Table 2).

The biomarkers for iron status (ferritin, sTfR, and Hb) were not consistently intercorrelated (Table 2). The relationship between ferritin and Hb was significant (*p* ≤ 0.05) and moderate in magnitude in females only, and the relationship between ferritin and sTfR in the composite sample and in males became non-significant (*p* ≥ 0.05) after the removal of outliers. However, the magnitudes of the intercorrelations among ferritin, sTfR, and Hb were mostly negligible. Therefore, each biomarker was examined separately.

**Fig. 1:**
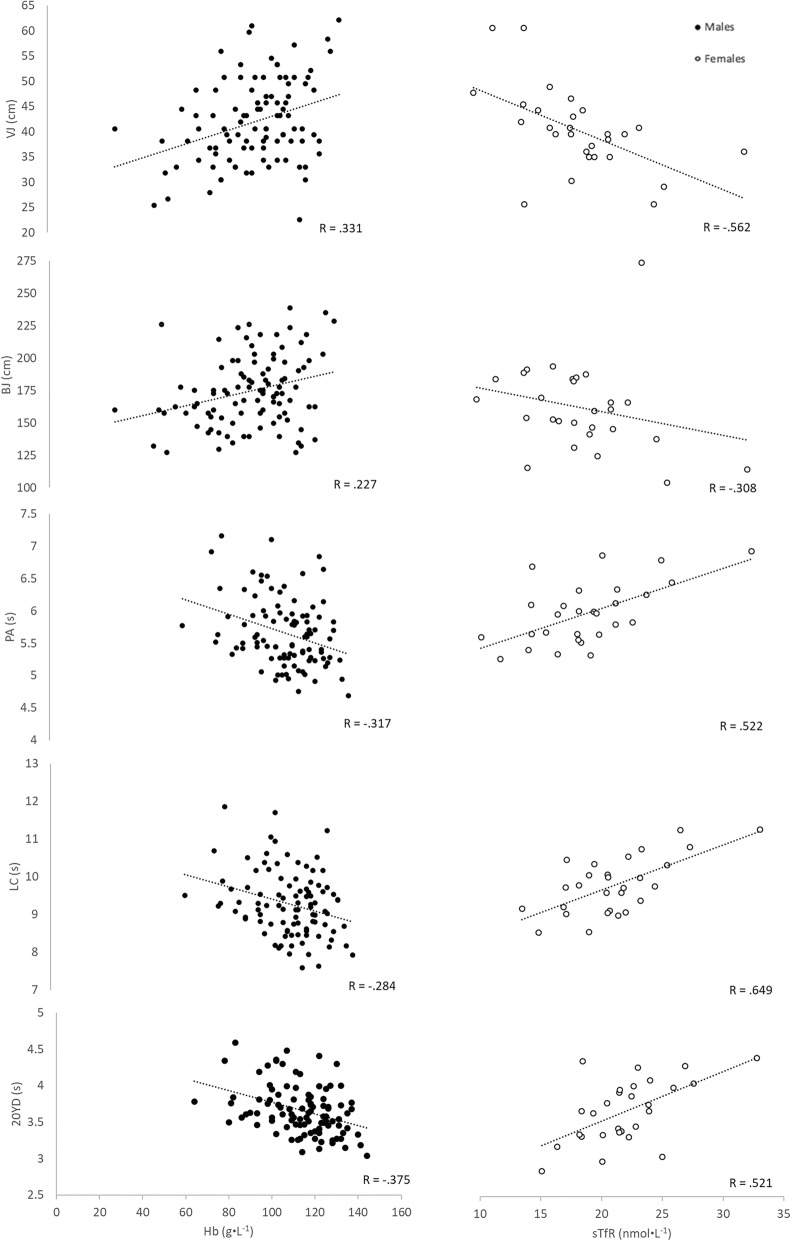
Scatterplots showing the sex separation of the relationships between athletic performance and biomarkers of iron status. Athletic performance was correlated with Hb in males (closed circles), while athletic performance was related to sTfR in females (open circles), both in the direction that was expected

Correlations among growth and development, athletic performance, dietary intake, ferritin, sTfR, and Hb are also illustrated in Table 2 and Fig. 1. Overall, growth and development was significantly (*p* < 0.001–0.048) correlated with athletic performance with magnitudes ranging from low to moderate. Age, maturity offset, and height exhibited nearly uniform, moderate correlations with athletic performance. Arm and thigh eCSA values were moderately correlated with VJ and BJ performances in males, while only thigh eCSA was moderately related to VJ and BJ performances in females. In males only, BF% exhibited moderate, inverse relationships with athletic performance, and PPU was moderately related to growth and development. Other significant (*p* ≤ 0.05) relationships among growth and development and athletic performance were low in magnitude.

Growth and development variables were not consistently related to dietary intakes, ferritin, sTfR, or Hb, with two exceptions. First, growth and development exhibited negligible, but significant (*p* = 0.004–0.042), relationships with protein intake in males. Second, growth and development displayed low to moderate relationships (*p* = 0.002–0.045) with iron intake in females.

Athletic performance was not consistently related to ferritin, sTfR, or Hb in the composite sample. However, when separated by sex, athletic performance exhibited consistent, negligible to low correlations with Hb in males (*p* < 0.001–0.05). Athletic performance also displayed consistent, low to moderate correlations with sTfR in females (*p* < 0.001–0.004). Figure 1 illustrates the relationships among athletic performance and Hb in the males (left scatterplots) as well as predominantly moderate correlations among athletic performance and sTfR in the females (right scatterplots).

Athletic performance exhibited consistent, negligible (*p* ≤ 0.05) relationships with energy and macronutrient intakes in the composite sample. When separated by sex, VJ and PA still displayed negligible relationships with energy and carbohydrate intake in males (*p* = 0.024–0.045). In females, athletic performance exhibited consistent, moderate correlations with iron intake (*p* = 0.001–0.027). Dietary intakes were unrelated to ferritin, sTfR, or Hb with mostly negligible correlations.

When focusing on the relationships among athletic performance, dietary intakes, and ferritin, sTfR, and Hb, partial correlations were calculated to remove the influence of concurrently related (possibly collinear) growth and development or dietary intake variables. In males, the partial correlations for Hb and athletic performance, while partialing out age and height were still significant for four of the six athletic performance tests: VJ, PA, LC, and 20YD (|*r*_Hb,y.Age_| = .208–.322, p = 0.001–0.041 and |*r*_Hb,y.Height_| = .211–.321, *p* = 0.001–0.038), respectively. After partialing out weight from the correlations between ferritin and three athletic performance tests (BJ, PA, and 20YD), the relationships were still significant (|*r*_Ferritin,y.Weight_| = .257–.360, *p* < 0.001–0.013). However, after partialing out BF%, the relationships between ferritin and athletic performance disappeared (|*r*_Ferritin,y.BF%_| = .035–.122, *p* > 0.05). Partial correlations in males for athletic performance and Hb after partialing out dietary intakes were also still significant (|*r*_Hb,y.energyintake_| = .369–.383, *p* = 0.005–0.007 and (|*r*_Hb,y.carbohydrate_| = .249–.368, *p* = 0.007–0.009). For females, the partial correlations in two athletic performance tests (PA and LC) with sTfR remained significant after partialing out iron intake (|*r*_sTfR,y.ironintake_| = .516–.569, *p* = 0.014–0.028), but the relationship between sTfR and the other performance tests (VJ and 20YD) were no longer significant (|*r*_sTfR,y.ironintake_| = .028–.460, *p* > 0.05).

## Discussion

The primary findings of the present study indicated that athletic performance was moderately related to sTfR concentrations in the female adolescent athletes, while athletic performance exhibited low correlations with Hb concentrations in the male pre-adolescent athletes (Fig. 1). Athletic performance was also moderately related to dietary iron intake in the females. Yet, conversely, there were no consistent relationships among dietary intakes, ferritin, sTfR, or Hb concentrations. Thus, dietary intake data was unable to track the biomarkers of iron status, but athletic performance, particularly in the female adolescent athletes, was directly proportional to sTfR and dietary iron intake. Although these correlations cannot infer causal relationships between sTfR or dietary iron intake and athletic performance, these findings can be hypothesis-generating.

Previous studies have established links between exercise performance and iron status in adult female athletes [9, 11, 12]. For example, active young adult females exhibited higher sTfR concentrations than sedentary females, but no other iron biomarkers were different between the two groups [12]. In female collegiate athletes, a positive relationship was reported between ferritin and VO_2peak_ [9]. The authors reported that a slower 4-km time trial performance was associated with iron depletion, but again no other relationships were observed with other iron status biomarkers [9]. Improvements in skeletal muscle strength were related to changes in Hb concentration following dietary iron supplementation in adult female elite volleyball players [11], but no other iron biomarker was related.

Interestingly, the adolescent female athletes in the present study demonstrated an inverse relationship between sTfR concentrations and athletic performance. That is, measures of athletic performance improved as sTfR concentrations decreased in the females (Table 2). Since sTfR is inversely proportional to iron availability [38, 39], which is thought to reflect erythropoiesis [39], red blood cell availability and function during athletic performance testing may be affected. It is possible that the iron availability in adolescent female athletes during their PHV may not be capable of supporting the demand for red blood cell production, thereby hindering skeletal muscle performance. Our findings may also tentatively suggest that the sTfR biomarker may be more sensitive than the other iron biomarkers in adolescent female athletes experiencing rapid growth, compared to adult athletic females.

Concentrations of Hb have also been associated with exercise and performance in young males [8, 10]. Cullumbine [8] reported low correlations between Hb and 100-yard sprint time (*r* = − 0.360) and deadlift strength (*r* = 0.440) in 14–20-year-old males. Gracia-Marco et al. [10] reported negligible, but significant, associations between Hb and BJ performance in 12.5–17.5-year-old males before (β = 0.286, *p* < 0.001) and after (β = 0.203, *p* = 0.001) covarying for age, seasonality, latitude, BMI, and moderate-to-vigorous physical activity level. The results of the present study demonstrated similar negligible to low correlations between Hb and VJ, BJ, PA, LC, 20YD and PPU in the pre-adolescent males (Fig. 1). The previous studies [8, 10] included older males (average age of 15 years) and reported higher average Hb concentrations (147 ± 12 and 151 ± 2 g·L^− 1^, respectively) than the present study. Furthermore, neither previous study measured or accounted for biological maturity or muscle mass. The uniqueness of the present study included younger males (Table 1), lower Hb concentrations (Table 1), no relationships between Hb and maturity offset or Hb and muscle mass (Table 2), and the partial correlations that removed the influences of age and height from the correlations between Hb and athletic performance. Our findings suggested that even after removing the influence of growth and development, the relationships between Hb and athletic performance were still significant in these pre-adolescent male athletes.

The presence of an association between Hb concentration and strength, speed, or power measurements suggests that Hb may influence anaerobic exercise performance. Given the oxygen-carrying capacity of Hb, relationships between Hb and aerobic fitness are expected and have been demonstrated in adults [40–42]. Since anaerobic exercise performance is theoretically independent of oxygen availability, relationships between Hb and anaerobic performance are more difficult to explain. Interestingly, all the athletic performance measures in the present study are anaerobic in nature, and many previous studies have demonstrated associations between anaerobic exercise performance and iron status [8, 10, 11, 43]. For example, the strength of association between Hb and BJ reported by Gracia-Marco et al. [10] was greater than the strength of association between Hb and cardiorespiratory fitness in the same sample (β = 0.192, *p* = 0.002). Potential physiological explanations may include the predominant, but not exclusive, anaerobic metabolism utilized, particular in children who rely more on oxidative mechanisms [16, 17, 44] and/or the oxygen-dependent resynthesis of creatine phosphate in the mitochondria [19, 20]. These relationships in children may also be impacted by a higher reliance on myoglobin-rich, oxidative fibers [18], allowing the oxygen carrying capacity of Hb to be more influential during anaerobic power, agility, and speed. Future studies are needed to test the hypotheses generated by the present and previous [8, 10] cross-sectional, correlational studies.

In an early study, Cullumbine [8] stated that “… males are faster than females and they have a greater strength at all ages; they also have consistently higher blood hemoglobin levels” (p. 276). Yet, the results of the present study did not entirely support the findings of Cullumbine [8]. In contrast to Cullumbine [8], there were no differences between the males and females in Hb or sTfR concentrations. When considering all measured variables, the largest sex differences were 32 to 40% greater upper-body strength (PPU) and muscle mass (arm eCSA), protein and iron dietary intakes, and ferritin concentrations. Moderate sex differences (10 to 18%) were evident in lower-body power (BJ and VJ), lower-body muscle mass (thigh eCSA), and energy and carbohydrate intakes. All other variables, including sprint speed (20YD), agility (PA and LC), fat intake, and Hb and sTfR concentrations were either equivalent or < 5% different between these young male and female athletes. Differences in upper-body, and to a lesser extent lower-body, strength and muscle mass are well-documented between boys and girls of this age [25, 26, 45]. Less is known about the dietary intakes and iron status biomarkers in relation to performance among this demographic. Since dietary intakes are reasonably modifiable, we would recommend increasing protein and iron intakes in young female athletes of this age. Future studies are needed to examine whether following such dietary recommendations results in improved ferritin concentrations and possibly athletic performance outcomes.

Despite the similarity in chronological age between the males and females in the present study, the females were experiencing a growth spurt (− 0.5 to + 0.5 years of maturity offset) at the time of data collection. In contrast, the males were 1.7 years away from their growth spurt (Table 1). This discrepancy between chronological age and biological maturity highlights the importance of interpretations involving growth and development. Previous research has hypothesized differences between young males and females in the timing of athletic development [25, 26], dietary needs and biomarkers of iron status [46]. The results of the present study extended existing knowledge by reporting relationships between growth and development and dietary iron intake in the adolescent female athletes, which was not observed in the pre-adolescent males (Table 2). Rossander-Hulthen and Hallberg [47] reported that starting at age 12, total estimated iron requirements increase in adolescent females, coinciding with the onset of menses. Adolescent females may need as much as 2.1 mg·d^− 1^ of dietary iron intake [47]. For comparison in adolescent males during their PHV, dietary iron requirements for the 50th percentile is approximately 1.8 mg·d^− 1^ [47]. However, the pre-adolescent males in the present study had not yet reached their growth spurt, which may explain why their dietary iron intake was not as related to growth and development as the females.

In contrast to dietary iron intake, dietary protein intake was related to growth and development in the males, but not the females in the present study (Table 2). Our findings supported those of previous studies [48, 49] related to protein intake and growth and development in young, growing males and females. Aerenhouts et al. [48] reported that on average, fat-free mass increased 2.44 kg·year^− 1^ and 3.84 kg·year^− 1^ in females and males, respectively, corresponding to protein accrual of 1.30 g·d^− 1^ in females and 2.04 g·d^− 1^ in males. These previous findings [48] suggest that the higher rate of skeletal muscle growth generally experienced in males may be associated with greater dietary protein needs for the younger, pre-adolescent males in the present study. Spear et al. [49] also suggested that protein needs of adolescents relate better to growth patterns than chronological age, especially in relation to height and tissue growth. Future studies may be needed to examine the relationships among growth and development measures and dietary protein intakes in males and females matched for biological maturity, rather than chronological age as is the case in the present study.

To further examine the relationships between athletic performance and Hb in males and sTfR in females, partial correlations were performed to see whether the relationships diminished after removing the influences of growth and development or dietary intakes. Neither growth and development (age and height) nor dietary intake (energy and carbohydrates) impacted the observed relationships between Hb and athletic performance. These findings suggest that Hb concentration is related to vertical power (VJ), agility (PA and LC), and speed (20YD) measures in pre-adolescent males, independent of growth and development or dietary intake. These findings, in conjunction with previous studies demonstrating relationships between Hb and anaerobic performance [8, 10, 11, 43], suggested that the oxygen-carrying role of Hb is at least partially related to anaerobic exercise performance. Since pre-adolescent children (only the males in the present study) tend to display type I muscle fiber characteristics [50], and type I fibers are heavily dependent on myoglobin [51], the associations between Hb and anaerobic exercise may be maturity-dependent. However, this hypothesis does not explain similar relationships observed between Hb and anaerobic performance in adults [11].

In addition, removing the influence of iron intake eliminated the relationships between sTfR concentrations and VJ and 20YD performance in the females. Therefore, iron intake at least partially explained the relationships between sTfR concentrations and athletic performance. This finding tentatively suggests that improving dietary iron intake could potentially improve athletic performance in adolescent females, particularly with regard to VJ and 20YD performance. Future studies are needed, however, to experimentally verify this hypothesis. The overall contrasting differences between the effects of partialling out collinear variables between males and females in the present study may have reflected differences in biological maturity, emphasizing the importance of maturity, rather than age, when monitoring diet and athletic performance in young athletes.

One limitation of the study is the initial recruitment of participants by age instead of maturity status. The study was designed to be field-test friendly to allow many young athletes to participate. The participants were recruited across the age range of 5–18 years old in order to be able to assess males and females falling into categories of pre-adolescent, adolescent, and post-adolescent. While categorizing by maturity status would be ideal due to the influence maturation has on iron requirements, hemoglobin levels, and athletic performance, this was not feasible for this particular study due to the recruitment and testing strategies utilized.

A potential limitation to this study was that only 39% of the total sample completed the online dietary recall. However, the correlations and partial correlations involving dietary intakes were performed with participants who displayed both values. According to the commonly-used table of critical values for correlation coefficients [52] using n-2 degrees of freedom and 5% type I error, the correlation coefficient that is considered statistically significant for the total sample in the present study is *r* = 0.195 (*n* = 249). The same critical correlation coefficient for only the participants who completed the dietary recall in the present study is still *r* = 0.195 (*n* = 97). These critical r-values indicate that the statistical interpretations of the composite correlation coefficients presented in Table 2, regardless of the smaller sample of dietary recalls, may be considered the same. Therefore, we believe that the smaller sample size of n = 97 for completed dietary recalls is still acceptable for addressing the research questions in this study.

Another potential limitation exists regarding sample size and the interpretations of iron biomarkers and dietary intakes for females. Since n = 24–31 samples were collected for iron biomarkers and dietary intakes, the critical r-values for these correlations are r = 0.349–0.423 [52]. However, we believe that the moderate correlations between sTfR concentrations and athletic performance, as well as the moderate correlations between athletic performance and dietary intakes, in the adolescent female athletes in the present study should not be ignored. Not only are children and adolescents a protected human subject population making it difficult to collect these data, but also adolescent female athletes may be considered an under-studied population. Together with the exploratory, correlational premise of the present study, we believe that these moderate correlations emphasize the need to collect additional data in adolescent female athletes in future studies to improve nutritional recommendations for this at-risk population.

## Conclusions

In conclusion, sTfR was moderately related to athletic performance (VJ, PA, LC, and 20YD) in the adolescent female athletes, possibly reflecting an increased rate of erythropoiesis during their growth spurt. However, after removing the collinear influence of dietary iron intake, relationships between sTfR and VJ and 20YD were eliminated, suggesting that improving dietary iron intake may improve lower-body power and speed in adolescent female athletes. The pre-adolescent male athletes showed significant, but negligible to low, relationships between Hb and athletic performance. After removing potential collinear influences of both growth and development (age and height) and dietary intakes (energy and carbohydrates), the relationships between Hb and athletic performance remained unaffected. From a more global perspective, perhaps the negligible to moderate correlations between iron status biomarkers (sTfR and Hb) and anaerobic performance in both male and female youth athletes reflect the subtle contributions of oxygen to exercise that is not exclusively anaerobic [53]. Interestingly, the fact that the adolescent females and pre-adolescent males exhibited different iron biomarker correlations, despite being at the same chronological age, suggested that iron status biomarkers may be more maturity- dependent than age-dependent. The largest differences between sexes in the present study included 32 to 40% greater upper-body strength (PPU) and muscle mass (arm eCSA), dietary protein and iron intakes, and ferritin concentrations for the young males. Based on these comparisons, we would recommend increasing dietary protein and iron intakes in young female athletes of this age. Nevertheless, these hypotheses need to be experimentally tested to clarify the underlying physiological relationships involving iron status biomarkers in pre-adolescent and adolescent athletes. Specifically, future studies should examine the effects of increasing dietary iron intake on ferritin, sTfR, and Hb concentrations, as well as athletic performance, in adolescent female athletes.

## Data Availability

The datasets used and analyzed for the current study will be made available from the corresponding author upon reasonable request.

## Abbreviations

20YD: 20-yard Dash
AGP: Alpha 1-acid Glycoprotein
BF%: Percent Body Fat
BJ: Broad Jump
eCSA: Estimated Cross-sectional Area
ELISA: Enzyme-linked Immunosorbent Assay
Hb: Hemoglobin
LC: L-cone
PA: Pro-agility
PARQ+: Physical Activity Readiness Questionnaire for Everyone
PPU: Power Push Up
sTfR: Soluble Transferrin Receptor
VJ: Vertical Jump
